# Nanofluidic-Based Accumulation of Antigens for Miniaturized Immunoassay

**DOI:** 10.3390/s20061615

**Published:** 2020-03-13

**Authors:** Denise Pezzuoli, Elena Angeli, Diego Repetto, Francesca Ferrera, Patrizia Guida, Giuseppe Firpo, Luca Repetto

**Affiliations:** 1 Department of Physics, University of Genoa, via Dodecaneso 33, 16146 Genoa, Italy; 2 Centre of Excellence for Biomedical Research, University of Genoa, viale Benedetto XV 9, 16132 Genoa, Italy

**Keywords:** nanofluidic device, immunoassay, antibody-antigen recognition, PDMS, miniaturized device, nanofunnel, immunobiosensing

## Abstract

The continuous advances of Nanofluidics have been stimulating the development of novel nanostructures and strategies to accumulate very diluted analytes, for implementing a new class of high sensitivity miniaturized polymeric sensors. We take advantage of the electrokinetic properties of these structures, which allow accumulating analytes inside asymmetric microfluidic structures to implement miniaturized sensors able to detect diluted solutions down to nearly 1.2 pg/mL. In particular, exploiting polydimethylsiloxane devices, fabricated by using the junction gap breakdown technique, we concentrate antigens inside a thin microfunnel functionalized with specific antibodies to favor the interaction and, if it is the case, the recognition between antigens in solution and antibodies anchored to the surface. The transduction mechanism consists in detecting the fluorescence signal of labeled avidin when it binds to biotinylated antigens. Here, we demonstrate that exploiting these electrokinetic phenomena, typical of nanofluidic structures, we succeeded in concentrating biomolecules in correspondence of a 1 pL sensing region, a strategy that grants to the device performance comparable to standard immunoassays.

## 1. Introduction

The interesting phenomena that take place when electric fields are applied to nanofluidic structures offer the possibility of improving the detection limits of conventional microfluidic platforms. In particular, Ion Concentration Polarization (ICP) has been intensively studied with the purpose of concentrating diluted analytes, as reported in recent reviews [1,2,3]. Generally, ICP is used to form a plug of concentrated analytes, which is then moved to the detection area, or to accumulate analytes directly on the sensing region. This second strategy results in being more appealing as it does not require auxiliary apparatuses for precisely handling fluids, but it implies that the structures for accumulation also have biochemical recognition capabilities. A typical approach for imparting biochemical selectivity to a structure consists in functionalizing its surface. This strategy has been extensively used in the field of solid-state nanopore/nanochannel-based sensors [4,5], as they allow combining the high sensitivity of the nanostructures—nanopores allow detecting single molecule translocations—with the selectivity provided by biochemical molecules immobilized on the surface. A variety of molecules have been immobilized on nanopores/nanochannels’ surface: amines [6], proteins and antibodies [7], DNA [8], synthetic nucleic acids [9], polyelectrolytes [10], polymers [11], and consequently, many functionalization strategies have been developed depending on the chemical properties of the material used for fabricating the nanostructure. Typical materials include semiconductor industry-derived materials (silicon, silicon nitride, silicon oxide, fused silica) and different kinds of polymers such as polyethylene terephthalate (PET), polymethylmethacrylate (PMMA) and polydimethylsiloxane (PDMS). It is now well-established that polymers, compared to hard-materials, offer many advantages in terms of costs and ease of fabrication; thus, they are widely used for the fabrication of sub-micrometric structures [12,13,14,15,16]. In particular, PDMS allows exploiting the simple and low-cost technique of replica molding (REM) for reproducing the sub-micrometric features of nanopatterned masters. Moreover, the chemical properties of PDMS, which can be easily tuned, make it a versatile substrate for the immobilization of biomolecules on its surface. In this paper, we show how it is possible to exploit functionalized PDMS nanofluidic structures to accumulate analytes and to detect their interactions with the molecules anchored on the surface. With this strategy, we succeeded in detecting biotinylated antigens with a concentration of nearly 1.2 pg/mL.

We took advantage of a novel fabrication technique [17] based on the junction gap breakdown approach, which allows overcoming the mechanical limits of PDMS when used for the fabrication of structures smaller than 100 nm. The large surface to volume ratio and the asymmetric geometry of these structures impart them a double function: accumulation and recognition, two key aspects for the development of miniaturized high sensitivity sensors. The first function is granted by the electrokinetic behavior of these devices, which show ionic current rectification characteristics [17], the latter by the functionalization of the surface of the device with biomolecules. In particular, we demonstrated, as a proof of concept, that functionalizing the device surface with anti-human IL10 antibodies, we can detect biotinylated human interleukin 10 (IL10) in very diluted solutions. We chose to work with this model as IL10 is a cytokine with powerful anti-inflammatory and immunosuppressive properties, whose levels are detected in many experimental disease models. 

To implement a miniaturized (nearly 1 pL) high sensitivity biosensor, we immobilized the anti-human interleukin 10 antibodies, on the PDMS surface; we accumulated biotinylated IL10 antigens inside the funnel, and finally, we detected the fluorescence signal at the funnel surface due to the binding of the fluorescent avidin with the biotin.

## 2. Materials and Methods

### 2.1. Fabrication of the Nanofunnel

**Figure 1:**
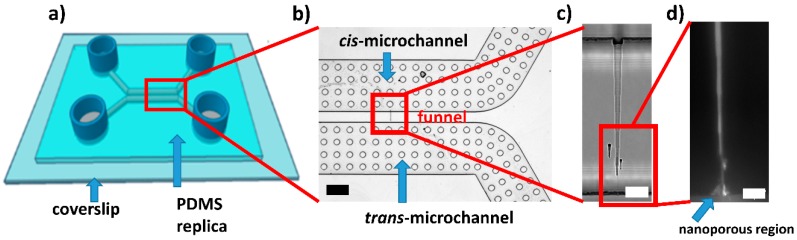
Polydimethylsiloxane (PDMS) miniaturized device: (**a**) schematic representation of the device consisting in a PDMS replica with two U-shaped microchannels (500 µm wide and 50 µm deep, separated by a gap 100 µm thick) sealed to a glass coverslip; (**b**) optical microscope caption of the *cis*- and *trans*- microchannels linked by a single funnel (highlighted by the red rectangle) (scale bar 100 µm); (**c**) bright-field caption of the entire funnel after the junction gap breakdown procedure (scale bar 10 µm); (**d**) epifluorescence microscope image of the nanoporous tip of the funnel, filled with a fluorescent solution, after the junction gap breakdown (scale bar 10 µm).

We fabricated the miniaturized nanofluidic devices replicating a nanopatterned silicon mold by using a double replica molding (REM) procedure. A detailed description of the fabrication process is reported in Angeli et al. [17]. Briefly, a focused ion beam (FIB) was used to pattern a tapered funnel-shaped structure linking two micromachined microchannels. The funnel, representing the only connection between *cis*- and *trans*- microchannels, is nearly 100 µm long, has a base around 7 µm wide and 4.5 µm deep, and a tip with an approximately triangular cross-section (nearly 800 nm wide and 80 nm deep). After the deposition of an anti-stiction layer of 1H,1H,2H,2H-perfluorooctyltrichlorosilane (FOTS) by vapor phase, a standard PDMS 10:1 (prepolymer:curing agent ratio w/w) negative replica was fabricated. After the same treatment, the negative replica was used as a mold to produce a composite PDMS positive replica of the micro/nanostructures patterned on the silicon mold, consisting of a thin PDMS 1:1 layer and a thick (a few millimeters) 10:1 layer. Then, after punching holes with a needle in each of the four reservoirs, the positive replica was exposed to an oxygen plasma treatment to obtain a watertight device (see Figure 1a). For overcoming the well-known problem of the “roof collapse” [18] that we experienced when sealing standard PDMS sub-100 nm structures with glass slides [19], we used a junction gap breakdown approach [17,20,21]. This approach leads to the fabrication of funnels with a nanoporous tip (Figure 1b–d), showing a rectifying behavior and the capability of accumulating analytes inside them. For the junction gap breakdown procedure, we used a sourcemeter (Sourcemeter 6478 by Keithley) and a couple of platinum wires. We inserted these electrodes into opposite reservoirs filled with 1 M KCl. This procedure exploits the dielectric breakdown of the collapsed PDMS at the funnel tip, for creating a nanoporous region, whose length depends on the collapse extent [17].

We purchased FOTS by Sigma-Aldrich, PDMS (DOWSIL™ 184 Silicone Elastomer Kit) (SYLGARD™ 184 Silicone Elastomer Kit) from Dow Chemicals. For the oxygen plasma treatment, we used a Tucano system (TUC-1B-MF, Gambetti Kenologia s.r.l., Italy).

### 2.2. Immobilization of Anti-IL10 Antibodies on the Funnel Walls

For functionalizing both the PDMS and the glass walls of a sealed device, we developed a procedure consisting of several steps. Initially, we exposed the device to an extended oxygen plasma treatment at 70 W, for 10 min, to introduce polar functional groups, mainly the silanol group (SiOH), which activates the surface and changes its properties from being hydrophobic to hydrophilic [22]. After activation, we inserted the device into a desiccator connected to a low vacuum pumping system and exposed it to vapors of (3-aminopropyl)triethoxysilane (APTES) for 5 min. APTES treatment was necessary because this molecule can bind to the hydroxyl functional groups of glass or activated PDMS at one end and has an amine functional group on the other end [23]. Subsequently, the microchannels were filled with glutaraldehyde (GA) in solution (2.5% *v/v* in deionized water) and placed on an agitator for 1 h at 25 °C to promote the diffusion of the solution. GA is a *bis*-aldehyde compound that has two reactive ends, which can crosslink two amine functional groups [23]. Therefore, GA was used as a linker between the APTES and the antibodies to immobilize them on the surface. After 1 h, the microchannels were washed with deionized (DI) water and with a phosphate-buffered saline (PBS) buffer 1X to remove the excess of GA. 

Then, a solution of mouse monoclonal anti-human interleukin-10 antibodies (anti IL10) in PBS 1X, with a concentration of 6.3 ng/mL, was inserted in the *cis*- microchannel (i.e., the microchannel connected to the tip of the funnel), while a PBS 1X solution was injected into the *trans*-microchannel (i.e., the one connected to the tip). The device, filled with these solutions, was placed on an agitator, for 2 h at 25 °C, to promote the homogeneous anchoring of antibodies on PDMS nanochannel walls. Then, microchannels were repeatedly washed with a PBS 1X buffer. At this point, we injected a solution of bovine serum albumin (BSA), 5% in weight in PBS 1X, into both the microchannels to “block” the non-specific binding sites of PDMS and glass surfaces. The device, filled with BSA, was placed on an agitator for 1 h. In the end, the excess of BSA was removed with repeated washing with PBS and DI water [23]. After this procedure, the device resulted in being biofunctionalized with antibodies and ready to be used for sensing experiments.

APTES, GA, PBS, and BSA were purchased from (Sigma Aldrich). Mouse monoclonal anti-human interleukin-10 antibodies (anti IL10) (ref. AHC8102) were purchased from Invitrogen (Thermo Fischer). 

The effectiveness of this procedure for biofunctionalizing PDMS was verified by acquiring infrared spectra of a slab of PDMS (radius of nearly 3 mm and 1 mm thick) at different stages of the process. We used a Fourier transform infrared (FTIR) spectrometer (FT/IR-4600 by Jasco, Japan) equipped with an attenuated total reflectance (ATR) accessory with a diamond crystal. For each spectrum, the sample was scanned 10 times in the range 500–4000 cm^−1^ at atmospheric pressure and room temperature. We pressed the PDMS slabs against the crystal with a standard screw.

### 2.3. Accumulation and Antibody–Antigen Recognition Experiments

For the antibody-antigen recognition experiments, we used solutions of matching and not-matching antigens for positive and negative tests, respectively.

In particular, we prepared solutions of matching antigens consisting of biotinylated recombinant human (rh) IL10 antigens (NF100 kit Fluorokine^®^ Biotinylated Human IL10, R&D system) diluted in PBS 1X at different concentrations (1.2 pg/mL, 12 pg/mL and 120 pg/mL) and of not-matching antigens, i.e., the negative control reagent provided by the NF100 kit (the soybean trypsin inhibitor biotinylated to the same degree as the cytokine). The concentration of the not-matching antigen solution was 5.0 ng/mL.

For accumulation experiments, we used the same set-up used for the dielectric breakdown procedure, consisting of Pt electrodes and a sourcemeter. In particular, to accumulate antigens and thus to increase the local concentration, a voltage bias was applied cyclically to the device filled with the solution of antigens. We performed preliminary tests for setting the parameters of the cycle (value and polarity of the voltage bias, duration of the time interval of application and resting phase), then, we fixed the parameters of the cycle to 10 min at −10 V and 10 min at 0 V. We repeated the cycle 3 times for each experiment. After the accumulation procedure, the antigen solution was removed, and a solution of avidin–fluorescein (avidin conjugated with fluorescein isothiocyanate 2.5 µg/mL at a ratio of 5:1) was inserted into the microchannels and shaken for 30 min at 25 °C protected from light. Each avidin molecule links five fluorescein isothiocyanate molecules. Avidin–fluorescein was part of the NF100 kit. Then, we repeatedly washed the microchannels with PBS 1X and DI water to remove unreacted fluorescent avidin. To verify if the binding between antibodies, immobilized on the surface, and antigens in solution occurred, we observed the funnel by using epifluorescence microscopy. In particular, we used a Nikon Eclipse Ti inverted microscope, equipped with a Zyla 5.5 camera by Andor, with a 60X λ-oil objective and a B-1E filter 540/20 nm. 

For investigating the accumulation mechanism itself, we performed experiments by observing the distribution of the fluorescence intensity signal produced by a solution of fluorescent avidin (30 pg/mL in PBS 1X) inside the funnel during repeated voltage bias cycles. To minimize photobleaching, we acquired images with a rate of 3.3 10^−2^ fps. 

## 3. Results and Discussion

### 3.1. Antibody Immobilization and ATR-FTIR Characterization

The goal of this study was demonstrating that nanofluidic structures, fabricated with low-cost approaches and materials, offer electrokinetic characteristics that can drive the development of a new class of high-sensitivity miniaturized biosensors.

**Figure 2:**
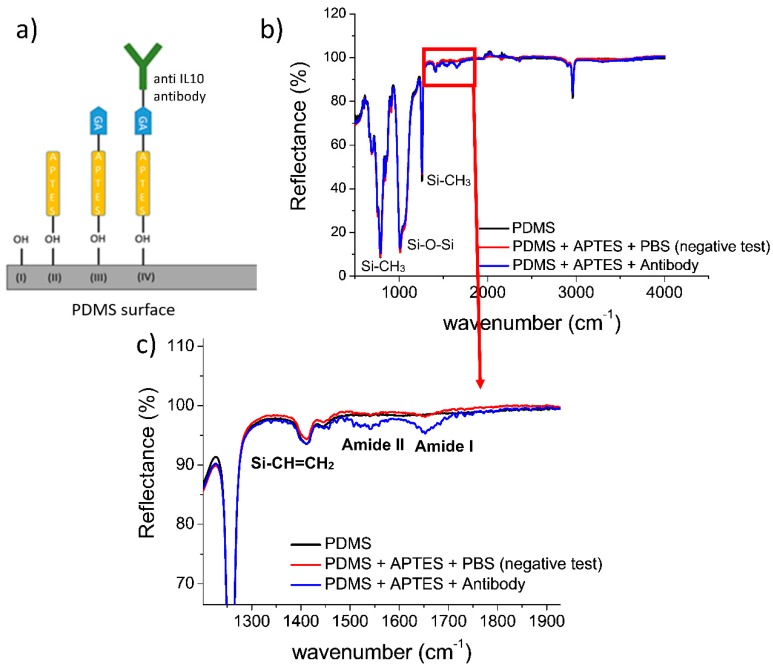
Antibody immobilization on PDMS: (**a**) scheme of the steps for functionalizing PDMS and glass with antibodies; (**b**) ATR-FTIR reflectance spectra acquired at different stages of the functionalization process; (**c**) detail of the reflectance ATR-FTIR spectra in the wavenumber range corresponding to amide I and II.

Taking advantage of the polymeric microfunnel–based devices, proposed by Angeli et al. [17], which showed the capabilities of accumulating analytes depending on the polarity and the intensity of the voltage bias, we fabricated the PDMS-based micro/nanofluidic devices by using the junction gap breakdown procedure. This procedure allows exploiting the advantages of the REM, a simple technique that does not require expensive equipment, and the versatility of PDMS, overcoming the limits of this material when used for fabricating sub-100 nm structures. Thanks to the junction gap breakdown, the PDMS funnels that typically experience problems of collapse, and thus result in being closed, are “re-opened”. In fact, after this procedure, a nanoporous network forms at the apex of the funnel. Figure 1 shows an example. This strategy allows distributing the fabrication costs of the master, which are high due to the use of nanopatterning tools, such as a FIB system, on a large number of polymeric copies produced starting from the same silicon master. The high potential of this fabrication approach and the innovative functionalities of these devices prompted us to explore applications in the field of biosensing. With this goal in mind, we developed a procedure for immobilizing antibodies on PDMS and glass, as these are the two materials used for the production of the sealed funnel. A scheme of the antibody immobilization strategy is reported in Figure 2a. Step (I) consists of exposing the sealed device walls, to an extended oxygen plasma treatment, for activating the surface of the PDMS microstructures, including the funnel. Then, we treated the device with APTES (step II), a widely used aminosilane, which is typically used to create surfaces exposing amine groups [24]. During step (III), we inserted GA into the microchannels, as this molecule can crosslink two amines, one on the surface and the other on the protein. Thus, step (IV) consisted of inserting a solution of anti-IL10 antibodies into the device to immobilize them at the microchannel surface. Then, the unreacted antibodies were removed from the microchannel by fluxing PBS 1X. To verify the effectiveness of the biofunctionalization process, we treated a thin slab of PDMS with the same protocol used for the device, and we checked the immobilization procedure comparing ATR-FTIR spectra acquired at different stages (see Figure 2). In detail, we measured: bare PDMS (black curve), oxygen plasma-treated PDMS exposed to APTES and PBS 1X (red curve), and PDMS exposed to all the functionalization steps, including antibody immobilization and washing (blue curve). 

The spectrum of bare PDMS shows negative peaks corresponding to specific functional groups; in particular, at 2965 and 843 cm^−1^ signals correspond to the stretching and rocking –CH_3_ groups, at 790 cm^−1^ to the Si–C stretching in Si–CH_3_ [25]. The peak at 1260 cm^−1^ is related to asymmetric C–H bending in SiCH_3_. The broad peaks between 1074–1020 cm^−1^ are typically related to the Si–O–Si stretching of the PDMS layer [26]. The main difference between the spectra is in the range of 1200–1900 cm^−1^, the blue spectrum shows a peak around 1640 cm^−1^, characteristic of amide I band with C–O stretching, and a peak around 1550 cm^−1^, corresponding to amide II band with C–N stretching and N–H bending [27]. These peaks, which are typically associated with the presence of proteins, were only detected on the PDMS functionalized with antibodies.

### 3.2. Accumulation and Antibody–Antigen Recognition

We used the functionalization strategy, described in the previous section, to immobilize probe antibodies on the PDMS surface of micro/nanofluidic devices fabricated by the junction gap breakdown approach.

**Figure 3:**
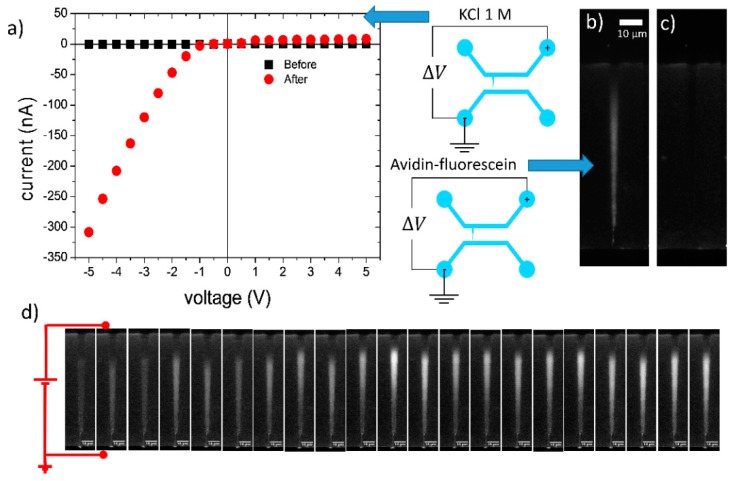
Accumulation experiments in devices functionalized with antibodies. (**a**) Current-voltage curve of a micro/nanofluidic device, filled with 1 M KCl, measured before (black squares), and after (red circles) the junction gap breakdown procedure. (**b**,**c**) Epifluorescence microscopy images of the funnel after 10 min at −10 V (b) and 10 V (c), with the *cis*-microchannel filled with avidin–fluorescein (12 pg/mL). (**d**) Sequence of epifluorescence images acquired at time intervals of 30 s during the application of a constant bias of −10 V for 10 min with the *cis*-microchannel filled by 30 pg/mL avidin–fluorescein.

**Figure 4:**
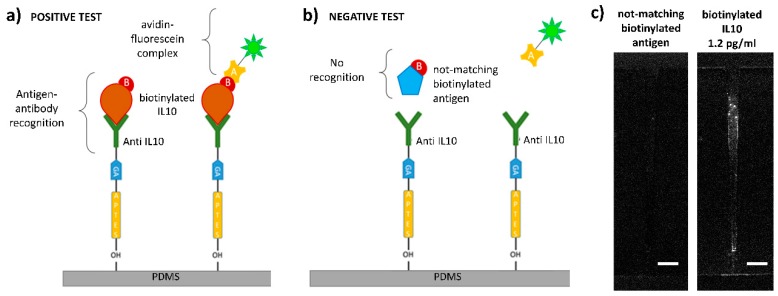
Antigen-antibody recognition mechanism and biosensing experiments. (**a**) Positive test: target biotinylated IL10 antigens in solution recognize and bind to the probe antibodies anchored to the surface; fluorescent avidin binds to the biotin, and it is detected by fluorescence microscopy; (**b**) negative test: biotinylated not-matching antigens do not bind with the probe antibodies, and they are washed away, no fluorescence signal can be detected. (**c**) Epifluorescence microscopy images of the funnel after a positive test performed with a biotinylated IL10 concentration of 1.2 pg/mL (right) and after a negative test (left).

Figure 3a shows a typical current-voltage curve of a device filled with 1 M KCl, acquired before and after the junction gap breakdown procedure. Analogously to the devices described by Angeli et al. [17], these micro/nanofluidic structures showed a clear rectifying behavior. Thus, to verify the accumulation capabilities of these devices, we performed accumulation experiments by inserting solutions of avidin–fluorescein in the *cis*-microchannel while applying a constant voltage bias. Similarly to our previous study [17], we analyzed the electrokinetic behavior of a functionalized device. We observed that avidin–fluorescein (12 pg/mL) in PBS 1X accumulates inside the funnel for negative bias, i.e., when the anode is on the tip side of the funnel (*trans*-microchannel) (see Figure 3b), while no fluorescence was detected for positive bias (see Figure 3c). Moreover, we monitored the accumulation process of a 30 pg/mL solution of avidin–fluorescein during the application of a constant bias of −10 V. We acquired epifluorescence images (1 s of exposure time) at time intervals of 30 s, to minimize photobleaching. We observed, in all the frames, a fluorescence signal inside the funnel, due to the local accumulation of avidin–fluorescein. Starting from these observations, we exploited the capabilities of the devices of concentrating analytes inside the funnel to favor the interaction between probe antibodies, anchored to the funnel surface, with target antigens diluted in solution. Then, we verified the biosensing capabilities of the device, performing positive and negative tests. The antibody-antigen recognition mechanism is depicted in Figure 4.

The positive test consisted of the following steps. We inserted a solution of biotinylated IL10 antigens (1.2 pg/mL, 12 pg/mL, or 120 pg/mL) in the *cis*-microchannel, then we applied a constant bias of −10 V for 10 min to accumulate target antigens inside the funnel to increase the local concentration. Then, we let the system at 0 V bias for 10 min. We repeated this cycle three times. Then, we washed away the solution of IL10 by fluxing PBS 1X, to remove unreacted antigens. We inserted a solution of avidin–fluorescein (diluted in PBS 1X 1:4) in the *cis*-microchannel, and we let it on a shaker, protected from light for 20 min. Avidin has a strong affinity for biotin. Thus, in the case of antibody-antigen recognition, the formation of the complex at the micro/nanostructure surface could be detected thanks to the fluorescence signal due to the fluorescent avidin linked to the biotin-conjugated antigen. Contrarily, for the negative test, we filled the *cis*-microchannel with a solution of biotinylated not-matching antigens and we applied the same electrical treatment and all the steps done for the positive test. Before the observation by epifluorescence microscopy, we thoroughly washed the microchannels with PBS 1X and DI water to remove the excess of avidin. We performed tests with 3 different concentrations on 6 devices. The results of an experiment, done by using a 1.2 pg/mL solution of matching antigens, are reported in Figure 4c. We compared fluorescence intensity signals, acquired with the same parameters (camera gain, exposure time, etc.), both for positive and negative tests, by subtracting images captured just after and before the tests, see Figure 4c. Only for the positive tests, we detected significant increases in the fluorescence intensity signal inside the funnel. It is worth noting that images in Figure 4c are displayed with the same brightness and contrast. We ascribed the fluorescence signal on the funnel walls to avidin linked to antigen-antibody complexes anchored to the micro/nanofunnel.

We detected a significant increase of the fluorescence signal, only when we applied the accumulation procedure to positive tests. Therefore, the concentration capabilities of the device resulted in being necessary for successfully detecting the antibody-antigen complex formation, especially when working with extremely diluted antigen solutions.

We measured the fluorescence intensity in different regions of the device but only in correspondence of the funnel we collected a significant signal. This phenomenon could be due to the high surface to volume ratio of the funnel compared to other regions, which makes it possible to detect such a low signal.

These experiments confirm that functionalized micro/nanostructures, used in combination with electrokinetic phenomena, typical of the nanoscale, can be exploited for the fabrication of miniaturized biosensors for detecting very diluted analytes.

## 4. Conclusions

Here, we demonstrated that PDMS biofunctionalized micro/nanostructures are promising platforms for the implementation of miniaturized and low-cost biosensors. We used biotinylated antigens from a commercial immunoassay kit as a proof of concept. By exploiting the ion concentration polarization phenomenon, typical of nanofluidic structures, we accumulated in a small volume target analytes to promote the interaction with probe molecules. This approach offers the advantage of combining concentration and detection in the same region, granting high sensitivity (up to 1.2 pg/mL) and selectivity to the device. We think that this approach, here demonstrated for a single funnel, can be easily parallelized for increasing the number of analytes and/or for enhancing the reliability of the sensor.
